# Zehn Jahre Bundesteilhabegesetz: Kontinuitäten, Konflikte und Perspektiven der Teilhabe

**DOI:** 10.1007/s00103-026-04269-9

**Published:** 2026-07-28

**Authors:** Matthias Morfeld, Uwe Koch-Gromus, Anke-Christine Saß

**Affiliations:** 1 Fachbereich Angewandte Humanwissenschaften, Hochschule Magdeburg-Stendal, Osterburger Straße 25, 39576 Stendal, Deutschland; 2 Institut und Poliklinik für Medizinische Psychologie, Universitätsklinikum Hamburg-Eppendorf, Martinistr. 52, 20246 Hamburg, Deutschland; 3 Abteilung für Epidemiologie und Gesundheitsmonitoring, Robert Koch-Institut, Gerichtstr. 27, 13347 Berlin, Deutschland

Vor 10 Jahren erschien im *Bundesgesundheitsblatt* ein Themenheft zu Behinderung und gesellschaftlicher Teilhabe^1^ zu einem Zeitpunkt, als das Bundesteilhabegesetz (BTHG) noch nicht verabschiedet war. Die Konturen waren aber bereits erkennbar – in der öffentlichen Debatte, in den fachlichen Reformdiskursen sowie in Forderungen von Verbänden, Wissenschaft und Praxis. Die Beiträge des damaligen Themenheftes nahmen Entwicklungen vorweg, die heute den rechtlichen und fachpolitischen Rahmen der Teilhabe bestimmen. Dazu gehörten seinerzeit die Abkehr von einer defizitorientierten Sicht auf Behinderung, die stärkere Orientierung am individuellen Bedarf, die Betonung von Selbstbestimmung sowie der Anspruch, Teilhabe nicht nur als Versorgungs-, sondern als Rechts- und Gerechtigkeitsfrage zu verstehen.

Das BTHG wurde 2016 beschlossen und bis 2023 in mehreren Stufen umgesetzt. Die Umsetzung lässt sich in unterschiedlichen Handlungsfeldern beobachten. Das gilt besonders dort, wo Reformen nicht nur rechtlich beschlossen, sondern organisatorisch, professionell und kulturell eingelöst werden müssen. Die Erfahrungen der vergangenen 10 Jahre verdeutlichen, dass gesetzliche Neujustierungen unverzichtbare Voraussetzungen schaffen, ihre Wirkung aber erst in der Praxis der Leistungserbringung, in den Zugängen zu Beratung und Unterstützung sowie in den institutionellen Kulturen sichtbar wird.

Das vorliegende Heft greift Fragestellungen von 2016 erneut auf und setzt neue Akzente. Mit dieser Verbindung von Kontinuität und Erneuerung hoffen wir, der gegenwärtigen Situation gerecht zu werden. Die Frage nach Inklusion und Teilhabe ist heute keineswegs erledigt; sie muss inzwischen allerdings differenzierter betrachtet werden. Teilhabe betrifft Rechtsansprüche und Leistungszugänge, aber ebenso die Qualität von Daten, barrierefreie Forschung, Partizipation in wissenschaftlichen und politischen Prozessen, Arbeits- und Bildungswelten sowie die Anerkennung von Menschen mit Behinderungen als Expertinnen und Experten ihrer Lebenslage.

Erklärtes Ziel des BTHG ist es, Menschen mit Behinderungen mehr Teilhabe und Selbstbestimmung zu ermöglichen und Leistungen stärker am persönlichen Bedarf auszurichten. In der Logik der Reform wurde Behinderung nicht länger ausschließlich als individuelles Defizit, sondern als Ergebnis einer Wechselwirkung von Beeinträchtigung und Umweltbarrieren verstanden. Dies bedeutete einen Perspektivwechsel: Die in der Behindertenpolitik und in der Disability-Forschung seit Jahren diskutierte Abkehr vom rein medizinischen Modell wird in den Rang einer sozial- und rechtspolitischen Leitlinie erhoben. Der Bericht des Bundestages zum Stand der Maßnahmen nach Artikel 25 Absatz 2 bis 4 des BTHG weist darauf hin, dass die Weiterentwicklung der Eingliederungshilfe in der Praxis noch nicht vollständig angekommen ist. Er macht zugleich deutlich, dass Reformen durch Daten, Evaluation, partizipative Forschung und fachliche Reflexion begleitet werden müssen.

Vor diesem Hintergrund versteht sich das nun vorliegende Themenheft als ein Dokument der politischen Zeitdiagnose. Es nimmt die damalige Reformdynamik auf, ohne sich darin zu erschöpfen, und fokussiert jene Fragen, die über den Zeitpunkt der Gesetzgebung hinaus Bestand haben. Besonders wichtig sind hier folgende Aspekte: Wer profitiert tatsächlich von den neuen Regelungen, welche Gruppen bleiben bei der Umsetzung unberücksichtigt und welche Indikatoren machen Teilhabe sichtbar? Zu klären ist außerdem, wie erreicht werden kann, dass Inklusion nicht auf formale Zugänglichkeit reduziert wird, sondern auch soziale, kulturelle und institutionelle Bedingungen einschließt.

Das Themenheft von 2016 enthielt unter anderem Beiträge zu Disability Studies, zum BTHG, zu Daten und Indikatoren, zur Teilhabeforschung, zu Barrieren in der medizinischen Versorgung sowie zu umwelt- und personbezogenen Faktoren. Damit verband das Themenheft rechts- und sozialpolitische Fragen mit methodischen, diagnostischen und versorgungsbezogenen Perspektiven. Diese Interdisziplinarität war notwendig: Teilhabe lässt sich nur dann angemessen verstehen, wenn sie nicht auf ein einzelnes System, eine Disziplin oder einen Indikator reduziert wird. Besonders hervorzuheben ist die Rolle jener Beiträge, die den Blick auf Daten und Messung lenkten. Sie sind für das Verständnis von Inklusion von zentraler Bedeutung, weil Teilhabe ohne geeignete Daten unsichtbar bleibt. Die damaligen Arbeiten zu Behinderung und Teilhabe in Deutschland, zu Empfehlungen der WHO, zu Perspektiven, die sich aus der Internationalen Klassifikation der Funktionsfähigkeit, Behinderung und Gesundheit (ICF) ergeben und zu Möglichkeiten der Gesundheitsberichterstattung bildeten die Grundlage für eine evidenzorientierte Diskussion, die heute noch trägt. Das aktuelle Themenheft knüpft daran an und benennt Forschungslücken, etwa im Bereich des Kindes- und Jugendalters, bei barrierearmen Erhebungen und bei standardisierten Teilhabeindikatoren. Damit wird deutlich: Wer Inklusion ernst nimmt, braucht nicht nur gute Absichten, sondern auch robuste, vergleichbare und sensible Daten.

Auch die damaligen Beiträge zu Versorgung, Rehabilitation und institutionellen Barrieren behalten ihre Aktualität. Das gilt ebenso für Beiträge, die sich mit Selbstbestimmung in spezifischen Lebensbereichen befassen. Sie belegen, dass Inklusion nur dann überzeugend ist, wenn sie auch dort gilt, wo persönliche Würde, Intimität und Selbstbestimmung berührt werden. Teilhabe ist immer an Zugänge, Ressourcen, Rechte und Kontexte gebunden. Behinderung ist nicht einfach eine Eigenschaft von Personen, sondern entsteht in Wechselwirkung mit Barrieren, Einstellungen, Regeln und Infrastrukturen. Damit wird Inklusion als Veränderung der Umwelt und nicht als Anpassungsleistung der Betroffenen verstanden.

Das vorliegende Themenheft verbindet zudem normative und empirische Perspektiven. Die Texte beschreiben nicht nur, was Teilhabe sein soll, sondern auch, wie sie erhoben, beobachtet und bewertet werden kann. Gerade in der Gesundheits- und Sozialberichterstattung ist das zentral, weil gesellschaftliche Teilhabe häufig erst politisch wirksam wird, wenn sie sichtbar gemacht werden kann. Das Heft ordnet Teilhabe in die Logik von Rechten ein und stellt zugleich Instrumente, Datenquellen und Forschungsperspektiven bereit, mit denen die Umsetzung des Anspruchs geprüft werden kann.

Darüber hinaus vermittelt das Heft Teilhabe als relationale Kategorie: Teilhabe bedeutet nicht nur Anwesenheit oder formale Teilnahme, sondern auch Beziehungsqualität, Zugang zu sozialen Räumen, Selbstvertretung und Beteiligung an Entscheidungen. Die hier versammelten Perspektiven zu Partizipation, Selbstbestimmung und institutioneller Praxis zeigen, dass Inklusion nur dann gelingt, wenn Menschen mit Behinderungen nicht Objekt von Fürsorge bleiben, sondern Subjekte ihrer Lebensgestaltung werden. Das ist auch der Maßstab, an dem sich das BTHG in seinem 10. Jahr messen lassen muss.

Welche der damaligen Hoffnungen wurden inzwischen zumindest ansatzweise eingelöst und wo stellen sich neue Aufgaben? Der Teilhabesurvey, neue Datenquellen, fortgesetzte Debatten um Barrierefreiheit und aktuelle Diskussionen über inklusive Forschung und Berichterstattung zeigen die Weiterentwicklung des Feldes. Zugleich ist die Umsetzung des Teilhabegedankens weiterhin stark von Strukturen, Ressourcen und professioneller Haltung abhängig. Die Beiträge in diesem Themenheft sollen helfen, Inklusion begrifflich zu klären, empirisch zu präzisieren und politisch einzuordnen; zugleich halten sie den Anspruch wach, dass Teilhabe mehr sein muss als ein Wort im Gesetz.

Liebe Leserinnen und Leser, wir hoffen, mit der Auswahl der Beiträge ein informatives Bild der aktuellen Diskussion zum Thema „Teilhabe“ zu vermitteln. Wir danken allen Autorinnen und Autoren der Beiträge sowie den Mitarbeiterinnen der Redaktion des *Bundesgesundheitsblattes* für die sehr gute Zusammenarbeit und wünschen Ihnen eine anregende Lektüre.

## Data Availability

Alle dieser Arbeit zugrunde liegenden Daten sind in diesem Artikel enthalten.

## Footnotes

1 Bundesgesundheitsblatt, Ausgabe 9/2016: https://link.springer.com/journal/103/volumes-and-issues/59-9.

